# AMD, an Automated Motif Discovery Tool Using Stepwise Refinement of Gapped Consensuses

**DOI:** 10.1371/journal.pone.0024576

**Published:** 2011-09-12

**Authors:** Jiantao Shi, Wentao Yang, Mingjie Chen, Yanzhi Du, Ji Zhang, Kankan Wang

**Affiliations:** 1 Key Laboratory of Stem Cell Biology, Institute of Health Sciences, Shanghai Institutes for Biological Sciences, Chinese Academy of Sciences, Shanghai, China; 2 Shanghai Institute of Hematology and Sino-French Center for Life Science and Genomics, Rui-Jin Hospital affiliated to Shanghai Jiao Tong University School of Medicine, Shanghai, China; 3 Graduate School of the Chinese Academy of Sciences, Shanghai, China; Louisiana State University Health Sciences Center, United States of America

## Abstract

Motif discovery is essential for deciphering regulatory codes from high throughput genomic data, such as those from ChIP-chip/seq experiments. However, there remains a lack of effective and efficient methods for the identification of long and gapped motifs in many relevant tools reported to date. We describe here an automated tool that allows for *de novo* discovery of transcription factor binding sites, regardless of whether the motifs are long or short, gapped or contiguous.

## Introduction

The regulation of gene expression is of critical importance for cellular activity and is controlled largely by transcription factors (TFs) [1]. TFs recognize short and complex DNA binding sites that are primarily located upstream of the transcription start site (TSS). Although experimental techniques play a definitive role in determining the functional binding sites, computational tools are becoming increasingly important for the prediction of transcription factor binding sites (TFBSs), particularly with respect to those present in the data obtained from global approaches such as expression microarrays, chromatin immunoprecipitation (ChIP) combined with DNA microarrays (ChIP-chip) or ChIP coupled with next generation sequencing (ChIP-seq).

However, the complexity and diversity of TFBSs necessitate continuing research in the area of computational prediction [2]. One of the major challenges in site prediction is the identification of short sequence patterns or motifs with statistical significance (or over-representation) in a given set of DNA sequences. These DNA sequences can be promoters of co-expressed genes or genomic regions targeted by a specific TF. The process of recognizing such motifs is defined as *de novo* motif discovery and is often performed without prior knowledge of the potential motifs to be discovered. Numerous *de novo* motif discovery tools have been recently developed and quickly adapted by investigators in the community, including AlignACE [3], REDUCE [4], MEME [5], YMF [6], MDscan [7], Weeder [8], DME [9] and Trawler [10]. Despite these available tools, the effective and efficient identification of motifs within datasets of interest remains a challenging problem, particularly when studying datasets derived from mammals, such as those from mice and humans. Tompa *et al*. [11] have recently evaluated 13 different motif discovery tools and showed that many of the tools are inefficient when used on datasets derived from organisms higher than yeast. For example, YMF was reported to be a more accurate tool than MEME or AlignACE for yeast data analysis, but its efficiency was significantly reduced when analyzing data from higher organisms, such as metazoans [12]. A recently developed tool, Amadeus [12], appears to outperform five other popular tools (AlignACE, YMF, MEME, Weeder and Trawler) on metazoan datasets, with a success rate of 62%. As indicated by the authors of Amadeus, the drawbacks associated with most of the other tools can probably be attributed to their selected background models because they are primarily based on pre-computed k-mer counts. In contrast, the reference background used in Amadeus includes the entire set of promoters in the genome of interest. This improvement appears to be especially important for the analysis of the genomes of organisms higher than yeast because their regulatory sequences are far more complex and versatile than the regulatory sequences in the yeast genome [12]. Other motif discovery tools improve performance by using discriminative algorithms, which take into account negative sets in the analysis. Members of this type of motif discovery tools include DME [13], DEME [14] and MoAn [15].

In addition, the algorithms used in most of the motif discovery tools are mainly designed to discover un-gapped motifs, which are usually less than 12 nt long. This restriction is largely due to the fact that motif discovery becomes more challenging when gaps are allowed [16] or when the motif length exceeds 12 nt [17]. However, the presence of gapped or long motifs is common in the genome of eukaryotes. According to the research by Xie et al. [18], up to 30% of the human motifs in promoter regions contain gaps. In their follow up research, hundreds of long motifs have been identified to be conserved across human, mouse and rat genomes. For example, the GAL4 motif is represented by the consensus sequence CGGnnnnnnnnnnnCCG. This type of motif is not easily identified by most motif discovery tools [10], [12]. Several specialized tools, such as SPACER [19], BIPAD [20] and SPACE [21], have been developed to search for such motifs. In addition, because long motifs are widely present in the eukaryotic genome and are often biologically indispensable, motif length is adjustable in several tools including MEME [5] and MDscan [7], which enables the use of these tools to identify long motifs. Unfortunately, low-effectiveness often accompanies these tools when searching for long motifs. Thus, there is a need to develop an automated approach for *de novo* motif discovery, regardless of whether the motifs to be discovered are gapped or un-gapped, or whether they are long or short.

Running time represents another important factor that impacts the process of motif discovery, particularly when dealing with ChIP-chip or ChIP-seq datasets, which usually contain thousands of binding regions for a single TF. For instance, MEME is one of the most popular tools used for these types of analysis but is computationally intensive when used on large target sets [10], [12]. For some other tools, running time appears to be determined by the motif length. The longer the motif to be discovered, the more running time required. This behavior is particularly true for enumeration-based tools such as Weeder [10]. Thus, the maximal length of motifs that can be efficiently identified by most tools is restricted to 12 nt [8], [10], [12]. However, many biologically significant motifs exceed this length [22].

In an attempt to address these challenges, we have considered multiple factors simultaneously, including the recognition of long or gapped motifs, accuracy of motif discovery and running time, and developed an automated motif discovery (AMD) approach. By comparing the performance of AMD with that of several of the most popular tools for *de novo* motif discovery, we found that AMD shared several of the advanced benefits of Amadeus, one of the most advanced motif discovery tools [12]. In addition, we found that AMD substantially overcame the drawbacks associated with Amadeus and all of the other tested algorithms in identifying gapped motifs and long motifs.

## Methods

**Figure 1:**
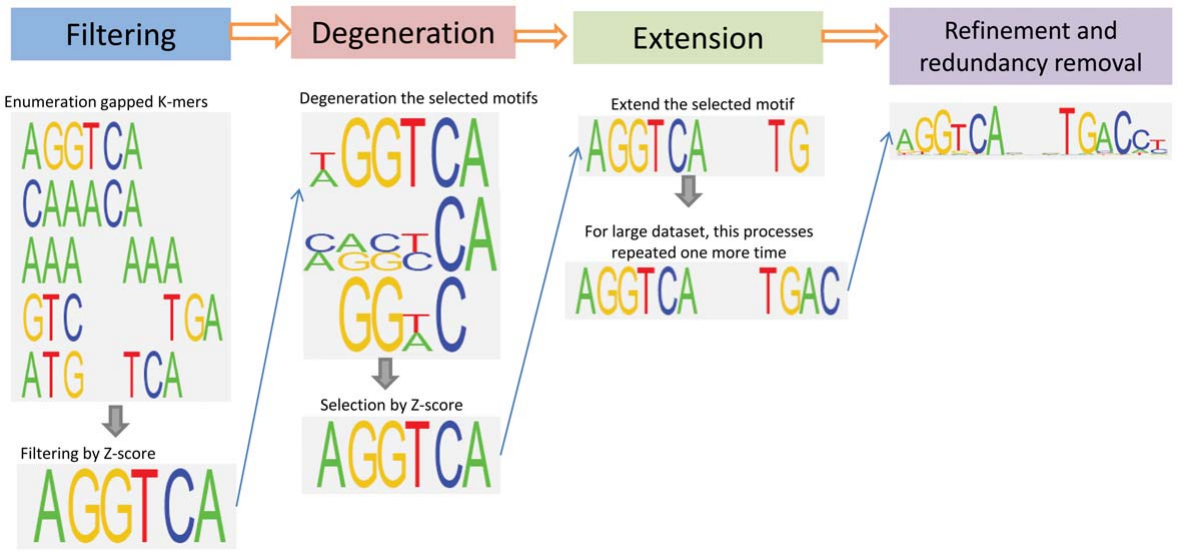
Overview of the AMD algorithm. AMD applies a step-wise motif discovery method, which includes core motif filtering, degeneration, extension, refinement and redundancy removal. The selected potential motifs in each step are subjected to the next step for further processing. For more details, see Methods.

We propose a *de novo* motif discovery method that identifies over-represented motifs in a group of foreground sequences compared to background sequences. The method is divided into five sequential steps: core motif filtering, degeneration, extension, refinement and redundancy removal. The selected potential motifs in each step are subjected to the next step for further processing. The foreground and background sequences are the only input files to the software. The main workflow is illustrated in Figure 1.

### Algorithm

Core motifs are represented by IUPAC consensuses that consist of two triplets of specified bases interrupted by a fixed number (from 0 to 14) of unspecified bases. For a given core motif, two scores are calculated by comparing the number of instances of the motif in the foreground and in the background:

- Fold enrichment: 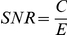 and

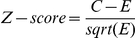
, quantifying the significance of enrichment [22].

Here, *C* and *E* represent the observed and expected number of instances in foreground regions assuming uniform distribution, respectively.

*E* is calculated as 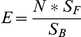, where *N* is the number of instances in the background, and 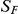 and 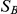 are the total length of the DNA sequences in the foreground and background, respectively.

Fold enrichments and Z-scores are used extensively in the first three steps.

#### Initial core motif filtering

An initial core motif is defined as UVW-gap-XYZ, where U, V, W, X, Y and Z can be any nucleotide. The gap length ranges from 0 to 14. This results in 61,440 (4^6^*15) potential core motifs. Thus, each initial core motif has a maximum length of 20 with 6 informative positions. The fold enrichment and Z-score for each of these core motifs are calculated. These core motifs are filtered by a minimal fold enrichment of 1.2, and ranked in descending order according to their Z-scores. The top 50 consensus sequences are selected as the primary core motifs. In case there are less than 50 consensuses left after fold enrichment filtering, all motifs are selected.

#### Core motif degeneration

In the second step, selected primary core motifs in the first step are updated to more degenerate ones. For each core motif, all possible motifs that are different from the original motif in at most 4 of the 6 positions are enumerated and all characters consistent with the initial character at that position in the core are tested, resulting in 983,040 (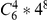) candidate core motifs. The motifs with fold enrichments and Z-score values greater than those of the primary core motif are selected as candidate degenerate core motifs. The most significant degenerate core motif (by Z-score) is chosen for each of the 50 consensus sequences selected in the first step. If no degenerate core motif is available, the original primary core motif is used for the extension step.

#### Core motif extension

In the third phase, the core motifs are extended. Equal numbers of non-informative N characters are added to each side of a core motif selected in the second step to obtain a core motif of 20 (for an even number of gaps between two three-mers in the original core motif) or 19 nucleotides (for an odd number of gaps between two three-mers in the original core motif). Then all possible motifs that are different from the original motif in at most 3 of the non-informative positions are enumerated. The resulting extended core motifs are evaluated and the motif with the largest Z-score is selected as the extended core motif if it has a higher Z-score than that of the un-extended form. For larger datasets (the genomic size of the foreground is larger than 100 kbps), this step is repeated one additional time. The 50 extended core motifs are retained for subsequent refinement.

#### Core motif refinement

Finally, we use the maximum a posteriori probability (MAP) to refine the 50 extended core motifs [7]. We scan all the instances of a given motif in the foreground with one mismatch allowed and use a modified MAP score to filter the candidate instances. The original MAP score used by MDscan [7] for a given motif is defined as the following:



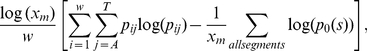
where *w* is the motif length, *x_m_* is the number of instances of the given motif in the foreground sequences, *p_ij_* is the frequency of nucleotide *j* at position *i*, and *p_0_(s)* is the probability of generating the sequence *s* from the background based on the third-order Markov model. The third Markov model is calculated based on the background sequences. For example, the probability of generating TCATG (assuming the three bases preceding this segment is AGG) from the background model is:
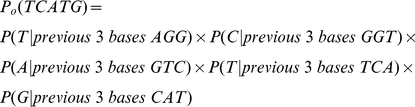



To account for the varied lengths of different motifs, *log(w)* is multiplied by the original MAP score.

The refinement procedure is similar with that in MDscan [7] and described below in brief. For a given extended motif, we scan all its instances in the foreground with one mismatch allowed. We firstly calculate the MAP score with all the motif instances identified using the equation defined above and donate it as 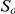. To test whether each of the identified instance is informative to the target motif, we then calculate the MAP score again by removing the tested instance and donate it as 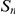. A instance is preserved if and only if 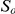 is greater than 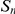. Finally all the preserved instances are merged to obtain a final motif that can be represented by position-specific weight matrix (PWM).

#### Redundancy removal

After the refinement step, the resulting 50 motifs are highly redundant, since similar motifs could be derived from different gapped consensus sequences. If the CompareACE score between two motifs is greater than a user-defined cutoff value, we only keep the motif with a higher MAP score, by a procedure as described below. We firstly sort the motifs by their MAP scores in a descending order. Then any motif that is similar (CompareACE score greater than 0.6, by default) with the highest ranking motif are removed from the list as redundant. The highest ranking motif is preserved and removed from the list, and then the second round of removal and selection is applied. Finally all the preserved motifs are reported.

### Measures of prediction accuracy

We adopted a set of performance measures based on the motif-level to evaluate motif discovery algorithms. For a given target set, we first assess the matrix similarity between the expected and the identified motifs using CompareACE [23]. When a cut-off is specified for CompareACE score, the success rate of a motif tool on a dataset can be calculated as the percentage of motifs correctly identified by a tool.

**Figure 2:**
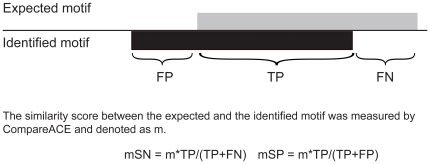
Motif-level measurements of prediction accuracy. The motif-level accuracy is evaluated by CompareACE scores and adjusted by factors determined based on the expected and identified motif lengths.

To take the motif length into consideration, two scores were developed to represent the motif-level sensitivity and specificity, as described below (Figure 2). The motif-level accuracy is evaluated mainly by CompareACE score and adjusted by a factor determined by the expected and identified motif length. Firstly the identified motif and expected motif were aligned using CompareACE. Then for each expected motif with identified motifs aligned, we then define the following values for calculating motif-level accuracy metrics: TP (true positive), the number of positions that common to the expected motif and identified motif; FP (false positive), the number of positions in the identified motif but not included in the expected motif; and FN (false negative), the number of positions in the expected motif but not in the identified motif.

The motif-level sensitivity of prediction accuracy (mSN) is defined as



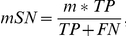
 and the motif-level specificity (mSP) is defined as



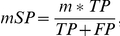



In both equations, *m* is CompareACE score between the expected motif and identified motif.

For each tool on each target set, the three top-scoring motifs were compared to the expected motif by CompareACE and the motif with the highest score was selected as the identified motif. The motif-level prediction accuracy scores for the target sets of the same group are averaged.

## Results

In this article, we present a *de novo* motif discovery tool, AMD, for the automatic identification of transcription factor binding sites. AMD has several crucial features. First, core motifs are represented by IUPAC consensuses [18] that consist of two triplets of specified bases interrupted by a fixed number (from 0 to 14) of unspecified bases (detailed descriptions are in Methods). This definition of core motifs should enable AMD to identify gapped motifs. Second, AMD can identify long motifs (both gapped and contiguous) because core motifs have a length of 20 nt with six informative positions and the potential to obtain more informative positions after the extension and refinement steps. Third, core motifs are updated in a stepwise procedure with a small number of candidate motifs evaluated at each step. This approach may significantly reduce the computational time needed for motif discovery. Finally, the background references are specifically designed according to the foreground sequences to be analyzed, increasing the efficiency and efficacy of motif discovery [12].

### AMD achieves a high success rate on both yeast and metazoan benchmark data

We first evaluated the motif discovery performance of AMD using two sets of well-characterized benchmark data from yeast and mammals. These sets were chosen because they have been widely adapted to evaluate the performance of *de novo* motif discovery tools [10], [12]. The yeast benchmark data were constructed from a large scale ChIP-chip analysis of yeast TFs across different biological conditions [24], and contains 230 target sets of 119 unique TFs. Sequences for all probes on the 6k microarray were used as the background. The PWMs of the expected motifs were retrieved from combined results from both computational analysis and literature which have been proved by the authors [24], [25]. The metazoan dataset included 32 mouse, human, worm or fly TF target collections derived from diverse high-throughput experiments [12], and the associated motifs were retrieved from TRNSFAC [26]. The promoter sequences of all genes in the genome were used as the background.

**Figure 3:**
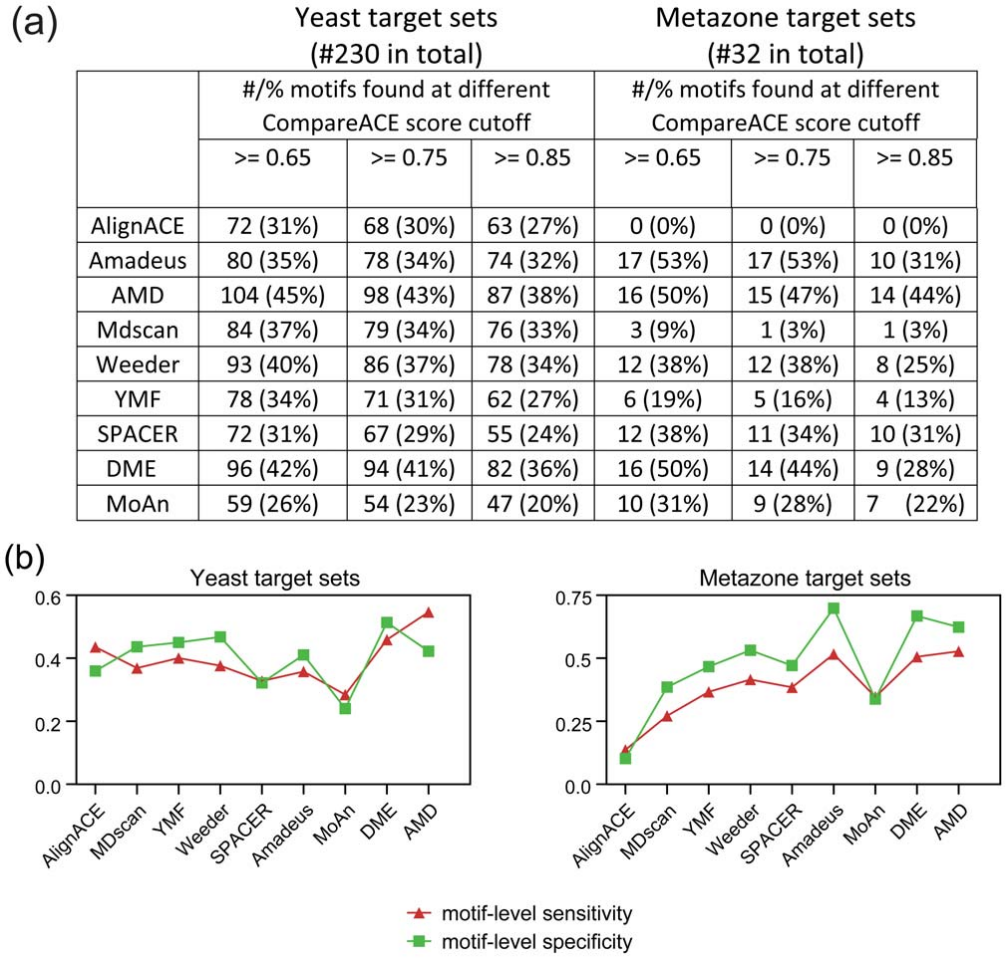
Evaluation of motif discovery tools on yeast and metazoan benchmarks. (a) Summary of the success rates of the tested algorithms on yeast and metazoan target sets. AMD and eight other tools were applied to the yeast and metazoan target sets. For each tool on each target set, the three top-scoring motifs were compared to the expected motif by CompareACE and the motif with the highest score was selected as the identified motif. Using a CompareACE score cutoff of 0.65, 0.75 or 0.85, the success rates of each tool on the yeast and metazoan target-set benchmarks were calculated. The number and percentage of motifs correctly identified by each tool are indicated. (b) Measurements of motif-level prediction accuracy of different tools on yeast and metazoan target sets.

We evaluated the seven most popular tools (AlignACE, MDscan, YMF, Weeder, DME, MoAn and Amadeus) and a gapped motif finder (SPACER) in parallel. To eliminate the potential bias introduced by the arbitrary cut-off, three CompareACE scores are used to summarize the success rate of each tool (Figure 3a). As it is shown, AMD performed the best on the yeast benchmark, although all tested methods performed similarly, with success rates ranging from 20% to 45%. As reported previously [11], each of the tested tools identified some unique motifs (Table S1). The performance of AMD was similar to that of Amadeus (a recently developed tool that is particularly suitable for motif discovery in metazoan datasets) and DME (a discriminative motif discovery tool that is known to typically outperform non-discriminative tools) on the metazoan benchmark tested (Figure 3a, Figure S1). In addition, we assessed the motif-level prediction accuracy of the various tools tested on the two datasets (details are in Methods) in a similar manner to a previously reported method [11]. As illustrated in Figure 3b, AMD, DME and Amadeus performed well on both the yeast and the metazoan datasets in terms of the motif-level sensitivity and specificity. AMD achieves the best performance on both two groups of target sets in terms of motif-level sensitivity (up to 0.6 for yeast and 0.5 for metazoan).

### AMD is effective at identifying both gapped and un-gapped motifs

We next selected an unbiased large dataset containing promoter collections of over 170 pre-defined motifs (57 gapped motifs and 116 un-gapped motifs) from the human genome [18]. These motifs were defined using an enumeration and conservation based approach, in which gapped consensuses were enumerated and filtered using a motif conservation score (MCS), followed by a clustering step [18]. These predefined motifs were used as the expected motifs in the evaluation. The promoter sequences (1 kb upstream to 200 bp downstream of the TSS) of the target genes of each motif were retrieved as target sets. The promoter sequences of all human genes were used as the background.

**Figure 4:**
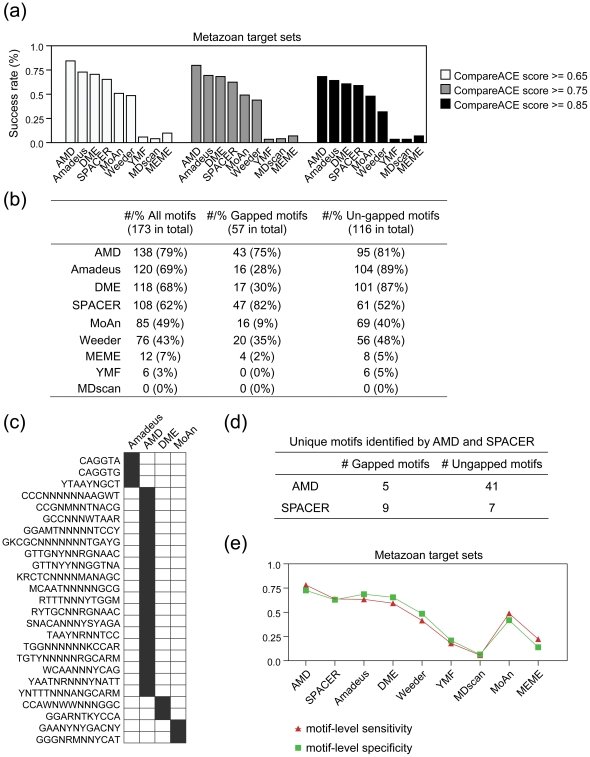
Assessment of the tested motif discovery algorithms on target sets of gapped and un-gapped motifs. (a) Summary of the success rates of the tested algorithms using different similarity cutoff. Using a CompareACE score cutoff of 0.65, 0.75 or 0.85, the success rates of each tool on the total target sets are shown. (b) Summary of the success rates of the tested algorithms on target sets of gapped and un-gapped motifs. All motifs were divided into two groups, gapped motifs or un-gapped motifs, based on the criteria of whether two or more continuous N characters were contained within the consensus sequence. Using a CompareACE score cutoff of 0.75, the success rates of each tool on the gapped and un-gapped motif target sets, were summarized. (c) Motifs successfully recovered only by Amadeus, AMD, MoAn or DME. Each successfully recovered motif is marked by a black-shaded box according to its CompareACE score (≥0.75). (d) Summary of the motifs specifically identified by AMD and SPACER. The numbers of gapped and un-gapped motifs specifically identified by AMD and SPACER are indicated. (e) Motif-level sensitivity and specificity of the nine tested tools on the target sets of gapped and un-gapped motifs.

We evaluated each of the tools mentioned above except AlignACE, since AlignACE was extremely computationally intensive and time-consuming when applied to the mammalian datasets (Table S2). As a classical motif discovery, MEME is also applied on this dataset for comparison. The success rate of each motif tool at three different CompareACE score cut-off is summarized in Figure 4a, which clearly indicates that AMD achieves the highest success rate regardless of cut-off used. For example at CompareACE score cut-off of 0.75, AMD achieved the highest success rate (79%) in terms of all the motifs tested, followed by Amadeus (69%), DME (68%), SPACER (62%), MoAn (49%), Weeder (43%), MEME (7%), YMF (3%) and MDscan (0%). When gapped and un-gapped motifs were counted separately, we found that AMD performed well on both motif types with success rates higher than 75%, whereas Amadeus and DME performed well only on un-gapped motifs and SPACER only on gapped motifs (Figure 4b). For example, in comparing the unique motifs identified by AMD with those identified by three other generic tools ranked second, third and fifth on the success rate list (i.e., Amadeus, DME and MoAn), AMD characteristically identified more gapped motifs (Figure 4c). Similarly, when AMD was compared to SPACER (a specifically designed gapped motif finder ranked fourth on the list), AMD identified significantly more un-gapped motifs than SPACER (Figure 4d). In addition, assessment of the motif-level sensitivity of each of the tested tools revealed similar results to those presented above (Figure 4e). Accordingly, we conclude that AMD represents a unified tool for the simultaneous recognition of both gapped and un-gapped motifs in data from real-life scenarios.

### AMD is highly sensitive to long motifs

A significant portion of biologically meaningful motifs exceed 12 nt in length and are defined as long motifs [22]. However, the maximum length of motifs discovered by many algorithms is restricted to a smaller number (usually less than 12) since the motif discovery process becomes very difficult due to the explosive growth in the number of possible variations as the motif length increases. To overcome this restriction, we updated the core motif in a stepwise manner, resulting in benefits in both time and memory efficiency for AMD. Furthermore, the core motif contained additional informative positions after the updating and refinement steps (see Methods), suggesting that AMD should be suitable for the identification of long contiguous motifs as well.

**Figure 5:**
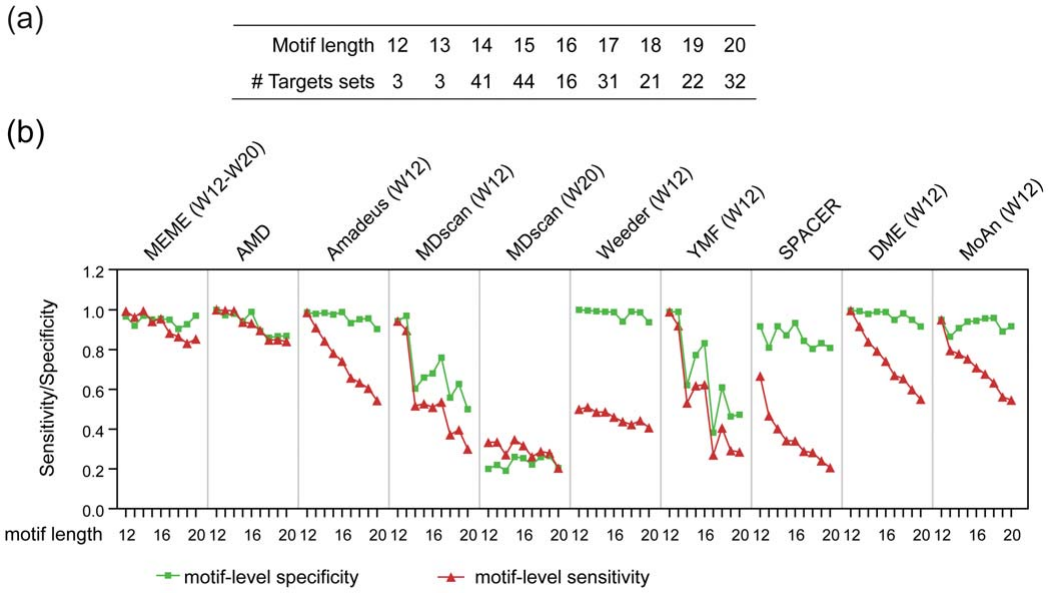
Performance of different motif tools on target sets of long motifs. (a) Summary of the numbers of target sets with different motif lengths. The 213 target sets of long motifs were divided into 9 groups according to the corresponding motif lengths. The number of target sets in each group is listed. (b) Performance of the nine motif tools on the target sets of long motifs at different lengths. Nine motif tools were applied to 9 groups of long motif target sets (as described in a). For each tool, prediction accuracy scores were averaged for target sets of the same group.

To test the performance of AMD in identifying long motifs, we constructed a long motif dataset containing target collections of 213 contiguous long motifs with lengths from 12 to 20 nt [22] (Figure 5a). These motifs were originally identified by enumeration of un-gapped k-mers (12 to 22 nt long) that were conserved in the human genome, followed by motif filtering and clustering [22]. The 1,000 bp sequences centered at the binding sites were used as a target set for each long motif. The background was selected from the sequences which were 500 bp flanking the binding sites of these long motifs.

To obtain the optimal results, the motif lengths were adjusted accordingly in several tools. The motif length for Amadeus and Weeder was set to 12 because this is the maximal motif length allowed. The motif length for MDscan was set to 12 and 20, respectively. To control the total running time with limited computational resources, the motif length for YMF, MoAn and DME were set to 12. For MEME, the minimal and maximal motif lengths are set to 12 and 20, respectively. AMD and SPACER both automatically selected significant motifs of the appropriate lengths.

As illustrated in Figure 5b, both AMD and MEME achieved high level of prediction accuracy in both the motif-level specificity (mSP ≥ 0.8) and motif-level sensitivity (mSN ≥0.8), indicating that they correctly captured most, if not all, of the informative positions of the long contiguous motifs in this dataset. MEME is a well-known tool that has good performance on discovery of long motifs and can be used as a positive control. It's noted that AMD, MEME and SPACER can find motif length automatically. All these tools achieved success rates, assessed by CompareACE at cut-off of 0.75, larger than 80%. However, SPACER achieves low motif-level sensitivity when motif length grows (0.2 for motif length 20). Although most of the other tested tools, including Amadeus and DME, achieved high motif-level specificity, none of them achieved motif-level sensitivity equivalent to that achieved by AMD, implicating that these tools captured fewer informative positions of the long motifs than AMD.

### AMD efficiently identifies long motifs in large datasets

Running time represents an important factor that affects the computational capacity of motif discovery tools, particularly when Gibbs sampling methods or enumeration-based tools are employed [10]. Although AMD is an enumeration-based method, we have integrated a procedure that updates core-motifs in a stepwise manner, resulting in a small number of candidates evaluated in each step. To validate whether this stepwise selection improved the computational capacity of our approach, we tested the running time of AMD and the six other tools on a defined panel of CTCF target collections with proportionally increasing numbers of target regions as described below. The significantly enriched regions of a large CTCF dataset [27] were ranked in descending order by their enrichment scores and the top scoring regions were selected to obtain 10 target sets with increasing numbers of sequences, ranging from 500 to 5,000 sequences. The background sequences were randomly selected from the genome using Cisgenome [28].

**Figure 6:**
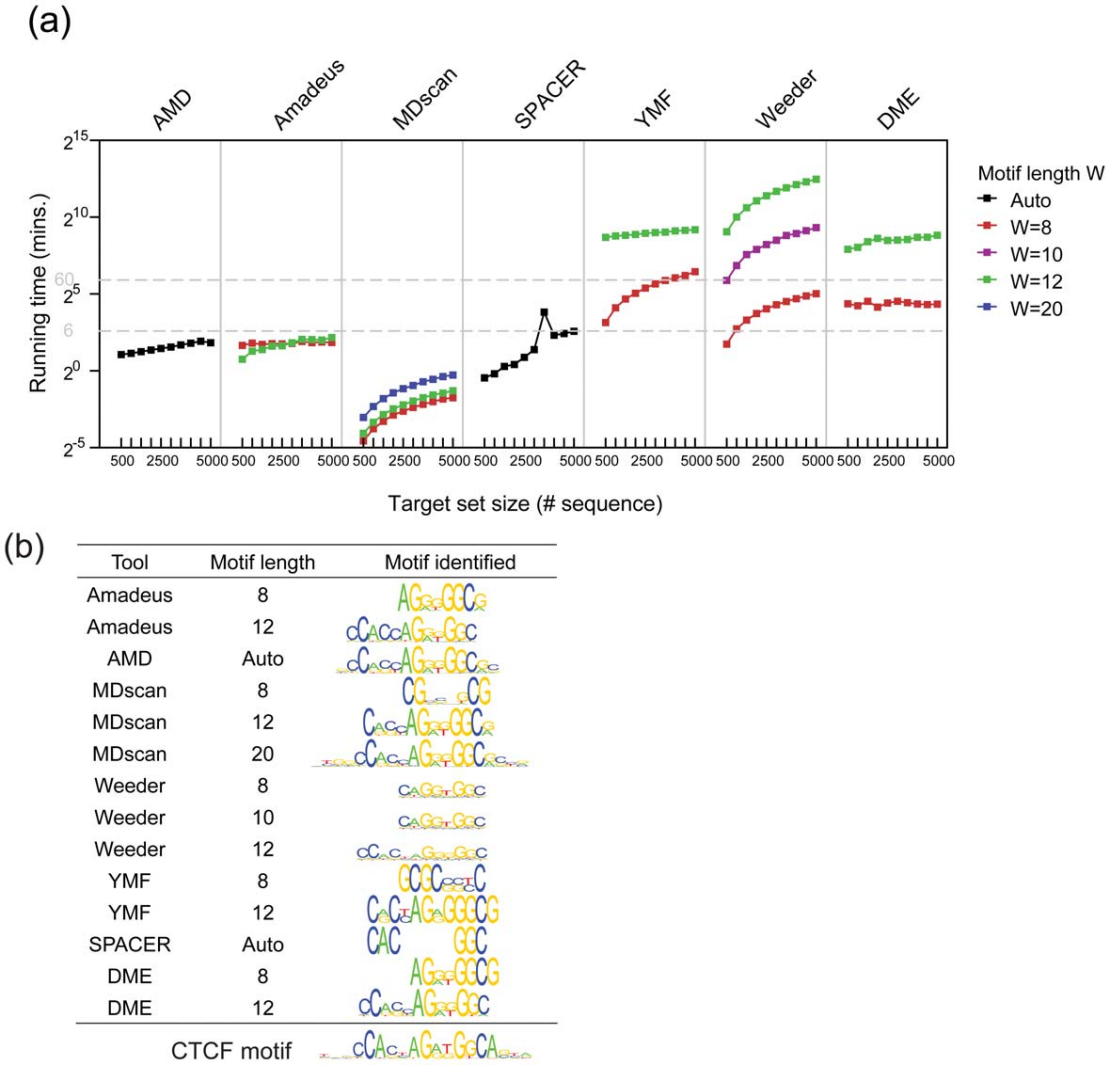
Running times of the tested tools in terms of the number of sequences in a dataset. (a) Running times for the CTCF target-sets with increasing dataset sizes on a logarithmic scale. Seven tools, including AMD, Amadeus (motif length W = 8, 12), MDscan (W = 8, 12 and 20), SPACER, YMF (W = 8, 12), DME (W = 8, 12) and Weeder (W = 8, 10, 12) were applied to the target sets with increasing numbers of sequences. The running times are plotted against the target set sizes on a logarithmic scale. (b) The logo of the representative motif identified by the tested tools. The motifs identified by each tool (with the different motif length settings) were compared to the expected CTCF motif, and the most similar motif was selected. Motif logos were generated with a local program that is a reimplementation of WebLogo (weblogo.berkeley.edu) in processing (www.processing.org). The setting of the motif length for each tool is indicated.

MoAn is not included in this part since it takes more than 3 days on each of these target sets (data not shown). As illustrated in Figure 6a, the typical enumeration based tool, Weeder, was the most time-consuming method. Further, as the length of the defined motif increased, the time required by DME and Weeder increased significantly (i.e., W = 8 vs. W = 12). The running times required by the improved enumeration based tools MDscan, AMD and Amadeus as well as the non-enumeration based SPACER, fell into a time window spanning only a few minutes. Of these tools, MDscan was the fastest tool. However, as shown in Figure 6b, AMD automatically identified 15 of 19 informative positions of the expected motif [22], Amadeus identified 12 of the positions at its maximal motif length setting (W = 12) and SPACER only identified a core motif with a few informative positions. Of note, it is common practice that multiple runs of data processing are needed for a given motif discovery tool to obtain more reliable results, as highlighted by the results obtained with Amadeus and DME (i.e., W = 8 vs. W = 12) in this setting, since the motifs to be discovered were usually unknown. However, AMD can automatically adapt its motif-length setting to obtain maximally matched informative positions in a single run. In this regard, the total running time of AMD would be much shorter than the time required performing multiple runs with other tools. This performance benefit is in addition to AMD's better ability to capture informative positions.

### Modeling TF binding using ChIP-chip/seq data

Recently, genome-wide ChIP-chip/seq analysis is increasingly used to identify *in vivo* binding regions of TFs [29], [30]. However, accurate modeling of actual TFBS on such high-throughput data remains a challenge. Accordingly, we tested AMD (in addition with Amadeus, DME, SPACER, Weeder and Trawler (web server) [10]) on an experimentally derived ChIP-chip/seq dataset containing target collections for seven TFs. Weeder is one of the most widely used tools for motif discovery in ChIP-chip/seq data. Amadeus and SPACER were included because they were effective tools for finding un-gapped motifs and gapped motifs, respectively, as described above. Trawler was included because it is claimed to be the fastest computational pipeline to date. As a discriminative motif discovery tool, DME was also included for comparison.

The seven ChIP-chip/seq target sets used to model the TFBS were constructed from publicly available datasets, which included SP1 (Affymetrix promoter array sample data) [31], PU.1 [32], FoxA1 [33], [34], Gli [35], ER [29], NRSF [30] and OCT4 [36]. Whenever possible, the most significantly enriched 1,000 regions from each dataset were selected for evaluation. The background for each target set was randomly retrieved from genomic sequences using packages in Cisgenome [28], which randomly select a matched control with similar statistics for each target set. The expected motifs were either retrieve from TRANSAFC database [26] (for SP1, PU.1 and Gli target sets) or publications (for FoxA1, ER, NRSF and OCT4 target sets).

**Figure 7:**
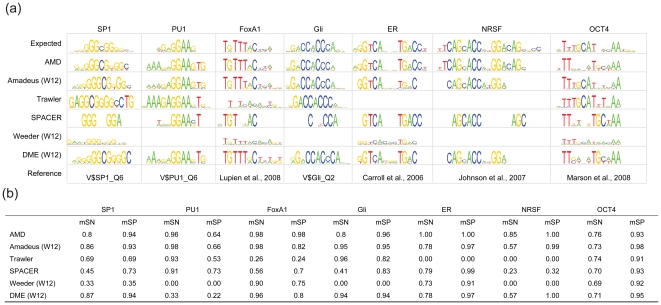
Modeling TFBSs using ChIP-chip/seq data. (a) A total of six selected motif tools were applied to seven ChIP-chip/seq datasets from humans and mice. The programs were either executed locally (AMD, Amadeus, DME, SPACER and Weeder) or via the Web server (Trawler). The expected motifs were retrieved from the TRANSFAC database or the literature (indicated in the reference row). The three top-scoring motifs were compared to the expected motifs by CompareACE and the most similar match is shown. An empty space indicates that no result was returned from the Trawler Web server or from Weeder. The generation of motif logos is illustrated in Figure 5. (b) Motif-level sensitivity (mSN) and specificity (mSP) of motif tools tested on ChIP-chip/seq datasets.

By comparing the findings from this analysis with the expected motifs (Figure 7a) using motif-level accuracy, we found that AMD performed similarly to Amadeus and DME on some motifs (SP1, PU.1, FoxA1, Gli, and OCT4), but significantly better on others (ER and NRSF). For example, AMD achieved motif-level sensitivity of 1.00 on ER dataset, while this value for Amadeus is 0.78 (Figure 7b). The better performance of AMD over Amadeus and DME on ChIP-seq data largely attribute to its ability to identify long motifs with the best length. Interestingly, although the PU.1 motifs identified by both AMD and Amadeus were highly similar to the expected PU.1 consensus, a more prominent string of adenosines at the 5′ of the GA core was recognized by AMD, which better supports experimental data regarding PU.1 binding [37].

## Discussion

The AMD software is an open source software and available from the google code [38] or Software S1. We have presented AMD as an automated discovery tool that allows for effective and efficient recognition of motifs in various datasets, regardless of whether these motifs are long or short, gapped or contiguous. The framework applied to AMD can likely be extended to other types of motif discovery pipelines as long as a proper objective function is defined, as described in detail below.

Sequence comparisons across different species may also represent an instrument to identify *cis*-regulatory elements [39]. In this regard, several *de novo* motif discovery tools have been developed and adapted to identify conserved motifs in yeast [40], fly [41] and human [18] genomes. Accordingly, we believe that the framework of AMD can be extended to identify this type of conserved motifs as well because these core motifs can be filtered, degenerated and extended using statistics such as MCS [18]. In the current implementation, AMD can effectively find motifs up to 20 nt in length, a size that appears to be sufficient for most currently known motifs [22]. It is therefore logical to expect that AMD could be applied to aligned sequences to identify conserved motifs, regardless of whether the motifs are long or short.

Like most other motif-finding tools, AMD identifies overrepresented motifs in a given foreground sequence set compared with a background sequence set. However, there are situations where multiple foreground sets should be considered simultaneously to identify biologically significant motifs. For example, in expression profiling analysis, genes can be assigned to different clusters by their expression patterns across different biological conditions [42]. Motif discovery in these clustered genes requires the consideration of multiple clusters simultaneously. The results from FIRE (finding informative regulatory elements) suggest that a motif can be informative not only due to its overrepresentation in a particular cluster but also to its under-representation in other clusters [43]. Under such circumstances, the integration of the strategy used in AMD may expand the scope of FIRE to accurately recognize complex motifs, such as long or gapped motifs.

## Supporting Information

Figure S1 **Evaluation of motif search tools on metazoan target sets.** Motifs identified by each tool were compared to the reference motifs using CompareACE. The results were shown in shadowed boxes as indicated.
(TIF)

Table S1 **The motifs specifically identified by each tool on the yeast data sets.** When the CompareACE score cut-off is set to 0.75, the motifs specifically identified by one tool are shown with a flag YES.
(DOC)

Table S2 **Running time of tested motif tools on mammalian target sets.**
(DOC)

Software S1 **The AMD software for Windows and Linux.** The compiled AMD software for Windows and Linux platforms are compressed in the RAR format. The demo dataset as well as the instruction document are also included.
(RAR)
