# Supplementary figures and images for: AMD, an Automated Motif Discovery Tool Using Stepwise Refinement of Gapped Consensuses
Source: PLoS One. 2011 Sep 12;6(9):e24576. doi: 10.1371/journal.pone.0024576 (PMC3171486; doi:10.1371/journal.pone.0024576)

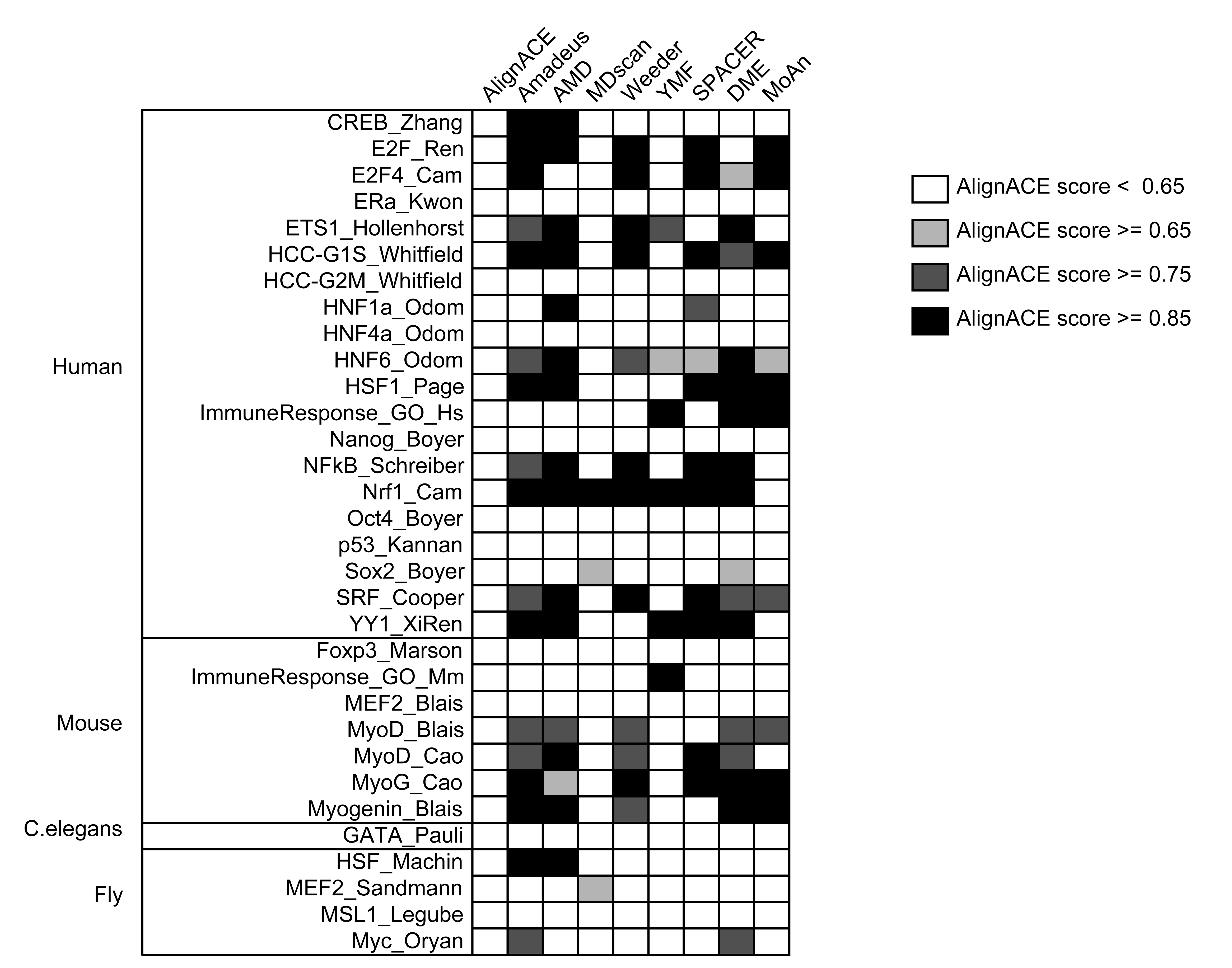

Supplement: Figure S1 — Evaluation of motif search tools on metazoan target sets. Motifs identified by each tool were compared to the reference motifs using CompareACE. The results were shown in shadowed boxes as indicated. (TIF) [file pone.0024576.s001.tif]
