# Supplementary material for: AMD, an Automated Motif Discovery Tool Using Stepwise Refinement of Gapped Consensuses
Source: PLoS One. 2011 Sep 12;6(9):e24576. doi: 10.1371/journal.pone.0024576 (PMC3171486; doi:10.1371/journal.pone.0024576)
Supplement: Table S1 — The motifs specifically identified by each tool on the yeast data sets. When the CompareACE score cut-off is set to 0.75, the motifs specifically identified by one tool are shown with a flag YES. (DOC) [file pone.0024576.s002.doc]

The motifs specifically recovered by one tool on yeast target sets

| Motif | AlignACE | Amadeus | AMD |  | Mdscan | Weeder | YMF | SPACER |  | DME |  | MoAn |
| --- | --- | --- | --- | --- | --- | --- | --- | --- | --- | --- | --- | --- |
| HAP2_YPD |  |  |  |  |  |  |  |  |  |  |  | YES |
| MET4_SM |  |  |  |  |  |  |  |  |  |  |  | YES |
| CST6_YPD |  |  |  |  |  |  |  |  |  | YES |  |  |
| MOT3_SM |  |  |  |  |  |  |  |  |  | YES |  |  |
| MSN2_Acid |  |  |  |  |  |  |  |  |  | YES |  |  |
| MSN4_Acid |  |  |  |  |  |  |  |  |  | YES |  |  |
| MSN4_H2O2Hi |  |  |  |  |  |  |  |  |  | YES |  |  |
| MSN4_H2O2Lo |  |  |  |  |  |  |  |  |  | YES |  |  |
| PDR1_YPD |  |  |  |  |  |  |  |  |  | YES |  |  |
| RCS1_YPD |  |  |  |  |  |  |  |  |  | YES |  |  |
| RME1_YPD |  |  |  |  |  |  |  |  |  | YES |  |  |
| SOK2_BUT14 |  |  |  |  |  |  |  |  |  | YES |  |  |
| SWI5_YPD |  |  |  |  |  |  |  |  |  | YES |  |  |
| XBP1_H2O2Lo |  |  |  |  |  |  |  |  |  | YES |  |  |
| IME1_H2O2Hi |  |  |  |  |  |  | YES |  |  |  |  |  |
| PDR3_YPD |  |  |  |  |  |  | YES |  |  |  |  |  |
| STP1_SM |  |  |  |  |  |  | YES |  |  |  |  |  |
| MET31_SM |  |  |  |  |  | YES |  |  |  |  |  |  |
| PHD1_BUT90 |  |  |  |  |  | YES |  |  |  |  |  |  |
| SPT2_YPD |  |  |  |  |  | YES |  |  |  |  |  |  |
| ARG81_YPD |  |  |  |  | YES |  |  |  |  |  |  |  |
| GLN3_YPD |  |  |  |  | YES |  |  |  |  |  |  |  |
| YAP6_YPD |  |  |  |  | YES |  |  |  |  |  |  |  |
| ACE2_YPD |  |  | YES |  |  |  |  |  |  |  |  |  |
| MATA1_YPD |  |  | YES |  |  |  |  |  |  |  |  |  |
| THI2_Thi- |  |  | YES |  |  |  |  |  |  |  |  |  |
| YML081W_YPD |  |  | YES |  |  |  |  |  |  |  |  |  |
| ROX1_H2O2Hi |  | YES |  |  |  |  |  |  |  |  |  |  |
| GCR2_SM | YES |  |  |  |  |  |  |  |  |  |  |  |

When the CompareACE score cut-off is set to 0.75, the motifs specifically identified by one tool are shown with a flag YES.
