# Supplementary material for: AMD, an Automated Motif Discovery Tool Using Stepwise Refinement of Gapped Consensuses
Source: PLoS One. 2011 Sep 12;6(9):e24576. doi: 10.1371/journal.pone.0024576 (PMC3171486; doi:10.1371/journal.pone.0024576)
Supplement: Table S2 — Running time of tested motif tools on mammalian target sets. (DOC) [file pone.0024576.s003.doc]

Running times of the tested tools on mammalian target sets (in minutes).

| Target sets | AMD | Amadeus | YMF | Mdscan | Weeder | AlignACE | SPACER |
| --- | --- | --- | --- | --- | --- | --- | --- |
| CREB_Zhang | 5.80 | 4.69 | 53.20 | 0.45 | 1242.23 | 4404.32 | 9.30 |
| E2F_Ren | 2.47 | 5.15 | 2.28 | 0.07 | 17.49 | 16.56 | 1.49 |
| E2F4_Cam | 2.61 | 4.80 | 4.37 | 0.09 | 51.73 | 91.19 | 2.50 |
| ERa_Kwon | 3.01 | 4.16 | 12.94 | 0.14 | 155.94 | 569.61 | 12.86 |
| ETS1_Hollenhorst | 3.78 | 4.31 | 24.02 | 0.26 | 528.84 | 2942.06 | 4.50 |
| HCC-G1S_Whitfield | 3.16 | 5.22 | 7.44 | 0.10 | 69.23 | 178.13 | 1.33 |
| HCC-G2M_Whitfield | 3.08 | 5.50 | 9.29 | 0.12 | 99.32 | 302.95 | 1.27 |
| HNF1a_Odom | 2.73 | 5.60 | 5.23 | 0.09 | 52.64 | 93.13 | 1.14 |
| HNF4a_Odom | 4.34 | 4.73 | 37.66 | 0.31 | 692.38 | 4404.33 | 5.89 |
| HNF6_Odom | 2.60 | 4.50 | 5.14 | 0.09 | 54.94 | 98.20 | 5.45 |
| HSF1_Page | 2.82 | 4.95 | 9.44 | 0.11 | 92.76 | 266.46 | 1.40 |
| ImmuneResponse_GO_Hs | 3.95 | 5.29 | 14.23 | 0.15 | 177.38 | 742.56 | 4.09 |
| Nanog_Boyer | 3.81 | 5.32 | 17.28 | 0.18 | 261.72 | 946.77 | 4.06 |
| NFkB_Schreiber | 3.00 | 4.91 | 7.83 | 0.10 | 72.87 | 175.90 | 1.80 |
| Nrf1_Cam | 3.49 | 4.02 | 14.84 | 0.17 | 247.27 | 1006.31 | 1.58 |
| Oct4_Boyer | 3.08 | 5.50 | 5.56 | 0.10 | 58.40 | 101.11 | 5.88 |
| p53_Kannan | 2.70 | 4.65 | 1.53 | 0.07 | 7.35 | 4.53 | 1.22 |
| Sox2_Boyer | 3.52 | 5.33 | 13.20 | 0.16 | 191.44 | 622.25 | 3.81 |
| SRF_Cooper | 2.88 | 5.28 | 4.44 | 0.08 | 39.82 | 66.67 | 1.80 |
| YY1_XiRen | 3.43 | 3.84 | 14.02 | 0.17 | 238.60 | 1077.63 | 1.61 |
| Foxp3_Marson | 5.18 | 5.79 | 21.73 | 0.22 | 446.94 | 1823.70 | 4.08 |
| ImmuneResponse_GO_Mm | 3.21 | 5.32 | 7.93 | 0.10 | 87.40 | 168.95 | 11.61 |
| MEF2_Blais | 2.71 | 4.61 | 1.11 | 0.05 | 4.75 | 1.24 | 0.95 |
| MyoD_Blais | 2.78 | 5.03 | 3.05 | 0.07 | 23.59 | 19.61 | 2.02 |
| MyoD_Cao | 3.05 | 4.79 | 3.22 | 0.07 | 23.64 | 20.88 | 2.57 |
| MyoG_Cao | 2.80 | 4.81 | 2.40 | 0.07 | 17.08 | 10.98 | 8.79 |
| Myogenin_Blais | 2.76 | 5.07 | 3.15 | 0.07 | 25.18 | 22.12 | 13.61 |
